# Factors That Influence Linkages to HIV Continuum of Care Services: Implications for Multi-Level Interventions

**DOI:** 10.3390/ijerph14111355

**Published:** 2017-11-07

**Authors:** Rogério M. Pinto, Susan S. Witte, Prema L. Filippone, Karen L. Baird, Wendy R. Whitman

**Affiliations:** 1 School of Social Work, University of Michigan—Ann Arbor, 1080 South University, Room 3792, Ann Arbor, MI 48109, USA; 2 School of Social Work, Columbia University, 1255 Amsterdam Avenue, 8th Floor, New York, NY 10027, USA; ssw12@columbia.edu (S.S.W.); plf2107@columbia.edu (P.L.F.); whitman.ma.lac@gmail.com (W.R.W.); 3 Department of Political Science, Purchase College, 735 Anderson Hill Road, Purchase, NY 10577, USA; Karen.Baird@purchase.edu

**Keywords:** HIV continuum of care, service providers, linkage to care, multi-level interventions

## Abstract

Worldwide, the human immunodeficiency virus (HIV) continuum of care involves health promotion providers (e.g., social workers and health educators) linking patients to medical personnel who provide HIV testing, primary care, and antiretroviral treatments. Regrettably, these life-saving linkages are not always made consistently and many patients are not retained in care. To design, test and implement effective interventions, we need to first identify key factors that may improve linkage-making. To help close this gap, we used in-depth interviews with 20 providers selected from a sample of 250 participants in a mixed-method longitudinal study conducted in New York City (2012–2017) in order to examine the implementation of HIV services for at-risk populations. Following a sociomedical framework, we identified provider-, interpersonal- and environmental-level factors that influence how providers engage patients in the care continuum by linking them to HIV testing, HIV care, and other support services. These factors occurred in four domains of reference: Providers’ Professional Knowledge Base; Providers’ Interprofessional Collaboration; Providers’ Work-Related Changes; and Best Practices in a Competitive Environment. Of particular importance, our findings show that a competitive environment and a fear of losing patients to other agencies may inhibit providers from engaging in linkage-making. Our results suggest relationships between factors within and across all four domains; we recommend interventions to modify factors in all domains for maximum effect toward improving care continuum linkage-making. Our findings may be applicable in different areas of the globe with high HIV prevalence.

## 1. Introduction

The World Health Organization (WHO) reports that, at the end of 2015, 36.7 million people worldwide were living with the human immunodeficiency virus (HIV). Sub-Saharan Africa remains the region most affected; it is home to 25.7 million (70%) of all those living with HIV worldwide [1]. The United States (US) Centers for Disease Control and Prevention (CDC) reported at the end of 2014 that nearly one million persons were living with HIV in the US [2]. The WHO recommends that individuals at risk worldwide be tested for HIV; if positive, they should initiate antiretroviral therapies (ART) [3]. ART lowers the viral load in the blood stream, making transmission of HIV less likely to occur [4,5,6,7]. Taken together, HIV testing, HIV care, and ART constitute the HIV continuum of care (“care continuum”)—recommended since 2012 as a preventive measure to reduce rates of HIV transmission worldwide. The care continuum engages at-risk individuals and those infected with HIV in a sequence of evidence-based services—HIV testing, primary HIV care, and ART—provided by trained medical personnel. Most individuals access these services through a diverse workforce of health promotion service providers (e.g., social workers, health educators, care navigators), who, in their day-to-day practices, establish and track linkages to medical personnel.

The extant literature does not have a widely agreed-upon empirical definition of “linkage” nor do organizations and practitioners abide by a common definition. Hereafter, we used the term “linkage” as it is used in practice by social and public health service providers in order to describe their attempts at “linking” clients to services (e.g., HIV testing) by, for example, phoning, emailing, or walking the client to meet another provider who can provide that service. The term “linkage” applies to each and every step of the care continuum; linkage to HIV testing, to HIV primary medical care leading to virus suppression, and also to support services that may be needed along the continuum. The term linkage here reflects our experiences as practitioners and the expertise of our Community Collaborative Board (ICCB) whose members make linkages in their day-to-day work (or supervise those who do) [8,9]. The CDC monitors the care continuum in two different ways. The prevalence-based approach defines the number of people at each step of the continuum as a percentage of the total number of people living with HIV (i.e., HIV prevalence). The diagnosis-based approach shows each step as a percentage of the number of individuals living with and who have been diagnosed with HIV. Data show that, in 2015, only 75% of persons receiving a diagnosis of HIV were linked to care within the one-month period defined as ideal by the CDC [2].

Successful entry in the care continuum can be facilitated through “engagement interventions” offered by service providers. In the course of providing counseling, substance use treatment, and other services, health promotion providers can help their clients to access HIV-related services [10,11,12,13,14]. Providers in myriad settings (e.g., community agencies, local health departments, health clinics, etc.) are well positioned to help patients access HIV testing and primary HIV care, adhere to ART, and stay in care [15,16]. Case managers and peer health navigators have been the foci of previous research [17,18,19,20,21]. However, in order to understand how best to reach patients inadequately engaged in care, a closer examination of how all providers can engage in interprofessional collaboration to help expand access to services is needed. Not every step in the care continuum depends on the previous step as an individual provider can not only link patients to care, but also help to retain them in care [22].

Gap in the literature. A robust body of research has shown that myriad patient-level factors influence linkages to care continuum services. Regrettably, at-risk and HIV-infected patients may not be receiving these services and life-saving care. Disparities in retention in HIV care and viral suppression have been identified among individuals facing untreated mental disorders, unstable living arrangements, or active substance abuse, and among people of color [21,23,24]. A major gap in the literature is research examining provider-, agency-, and policy-level factors that may also affect linkage-making [25,26] and also retention in care and viral suppression. There is a call for research to determine sociomedical factors (at the provider, agency, and policy level) that may influence linkage-making [23,24]. Therefore, these factors are the foci of this paper.

### 1.1. Conceptual Foundation

**Figure 1:**
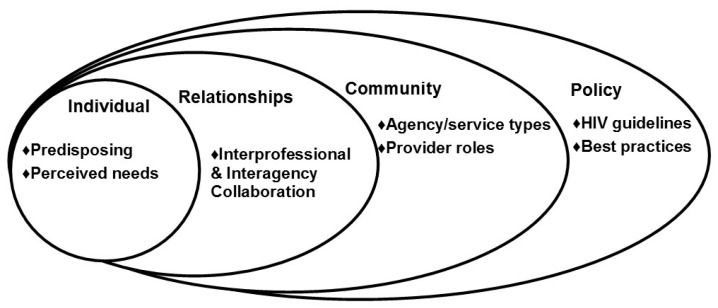
Socioecological influences on human immunodeficiency virus (HIV) continuum of care.

In order to improve linkage-making, we need to identify the key factors that influence linkage-making and then develop interventions to tackle provider-, agency- and policy-level barriers. To accomplish this aim, we conducted in-depth interviews with providers who participated in a longitudinal study examining the implementation of HIV services in 36 agencies. Drawing on a socioecological perspective [14], we uncovered provider-, interpersonal-, and agency-level factors that may influence linkage-making. We organized these factors around a framework (Figure 1) depicting how providers engage and retain patients in the care continuum. Our framework is a modified version of Mugavero et al.’s (2013) perspective and contains four domains of reference: Individual (provider), Relationships (interprofessional), Community (agency), and Policy (best practices). Though the original framework focuses on patients, we assert that factors in each domain also influence providers as they help patients through the continuum. For the purpose of this study, the Individual domain includes predisposing and enabling factors regarding providers (knowledge base and demographic characteristics). The Relationships domain includes interpersonal characteristics and capacities (social network, trustworthiness and communication). The Community domain comprises structural and functional factors of healthcare systems, including the type of and size/capacity of agencies that participated in the parent study. The Policy domain includes governmental and health organizations guidelines, such as best HIV prevention practices.

### 1.2. Factors that Influence Linkage-Making

Social and public health service providers, such as social workers, health educators, and care navigators, report that linkage-making is negatively influenced by factors in both the Community and Policy domains, such as work environments with insufficient budgets, physical space, and technological resources (internet access) [27,28]. Factors in the Relationship domain have been revealed in research on hospice, nursing, and other settings, suggesting that interprofessional collaboration can improve linkage-making across healthcare systems [29,30,31]. The literature also suggests that myriad Individual domain factors may affect linkage-making. For example, burdensome caseloads [32] and providers’ fear of losing patients to competing agencies may discourage linkage-making. These challenges can be exacerbated by providers’ professional fluctuations (job changes) and diminish their linkage-making and retention capacities [33]. Research also suggests that providers trained in linkage-making are best able to link patients to primary care, and help navigate and retain patients in medical systems [34,35]. Providers who are trained in evidence-based practices and/or hold positive attitudes toward them are more likely to adopt care continuum services [36,37,38,39]. In this paper, we identify salient factors that can improve and/or hinder linkage-making specifically in the context of the care continuum.

## 2. Materials and Methods

Data for this study came from a parent mixed-method longitudinal study, funded by the United States National Institute of Mental Health (R01MH095676), aiming to examine 250 providers’ implementation of HIV services in New York City. The study was approved by the appropriate Institutional Review Boards (University of Michigan # HUM00104278, Columbia University # IRB-AAAJ3058). Here we focus on the qualitative portion of the study.

### 2.1. Overview of Procedures for the Longitudinal Study

We recruited agencies that had funding earmarked for services targeting populations at risk for HIV infection. Study staff contacted agency representatives by phone, and provided information on procedures and inclusion criteria. We enrolled the first 36 agencies whose representatives accepted our terms. Each agency received a computer (valued at $1000) as an incentive. Representatives recruited providers who offered direct services, recruited patients, and/or delivered HIV services. The average number of providers per agency was 10 (2 to 25). Providers received $20 and $30 gift cards upon completion of baseline and 12-month follow-up interviews. Project staff implemented computer-assisted, face-to-face surveys (45–60). The survey included questions about implementation of HIV services, job descriptions, and demographics. A qualitative component of the study, the focus of the paper, inquired about provider linkage-making experiences and collaborative behavior (below).

### 2.2. Criterion Sampling Strategy for In-Depth Interviews

We used criterion sampling to focus on a pre-defined group of interest [40,41]: out of 250 participants in the longitudinal study, we selected 20 for in-depth interviews. Out of 250, 60 met criteria for the interview. We based our selection on their scores on two variables in the survey above: (1) frequency of linkages to HIV services (e.g., HIV testing, primary care, etc.); and (2) provider opinions/experiences with interprofessional collaboration. Our goal was to capture groups of providers exhibiting different patterns of linkage-making and levels of collaboration. We created four groups: “low” and “high” linkage-makers; and “low” and “high” interprofessional collaborators. Providers were than randomly selected from each of the four groups until five providers per group were represented in the sample (*n* = 20).

*Frequency of linkages to HIV and support services*. To determine the scores of type and frequency of linkages made in the past six months, we asked, “How often have you linked patients in the past six months”? We provided a list of 11 HIV-related and support services related to the care continuum: HIV, Sexually Transmitted Infections (STI), and Hepatitis-C (HEP-C) testing, primary care, mental health, housing, substance abuse, and syringe exchange services, public assistance, legal services, and vocational/educational services. These categorical responses were recoded to reflect the approximate number of linkages made in the past six months as follows: “no referrals” = 0; “once a month” = 6; “once a week” = 24; and “several times a week” = 48. The composite total number of linkages was computed as the sum of linkages across all 11 services. The composite total number of linkages was used to categorize providers into “low” (0–48) and “high” (>144) groups. We then used the 25th and 75th percentiles of the total number as cutoffs to categorize providers into “low” (0–48 linkages) and “high” (>144 linkages) linkage-makers, respectively.

*Provider opinions/experiences with interprofessional collaboration*. We evaluated interprofessional collaboration using the 49-item Index of Interdisciplinary Collaboration [42]. Participants self-reported the degree to which they agreed (1 = “strongly disagree” to 6 = “strongly agree”) with statements concerning five different areas of collaboration—interdependence, flexibility, professional activities, ownership of goals, and reflection on process. We used the 25th and 75th percentiles of the overall mean score as cutoffs to categorize providers into “low” (1.0–4.0) and “high” (4.3–6.0) interprofessional collaborators. This technique allowed us to create a more representative sample exhibiting different degrees of linkage-making and interprofessional collaboration.

### 2.3. Recruitment for In-Depth Interviews

All participants in the longitudinal study were informed about the possibility of being contacted for in-depth interviews. Randomly selected providers, whose data match the sampling criteria, were contacted for interviews. The first 20 providers who we contacted agreed to participate.

### 2.4. Interview Protocol and Data Collection

We used semi-structured questions to identify and contextualize factors that influenced how providers helped patients navigate the care continuum. Grounded in the socioecological framework, we asked participants about linkage-making, professional knowledge base, opinions, attitudes, and social norms around collaboration, and evidence-based practices. The interview started with a rapport-forming question, “How has your job been for the last 6 months”? Participants were asked to describe changes in their lives/jobs affecting their work. Two master´s-level interviewers, supervised/trained by the first author, conducted all interviews. Interviews (45–60 min) were digitally recorded and transcribed for entry into NVivo 11 software for managing, retrieving, and coding qualitative data [43]. Two independent researchers reviewed randomly selected recordings of six interviews and provided feedback to interviewers.

### 2.5. Analytic Approach and Data Interpretation

The two interviewers (the fourth and fifth authors) served as data analysts. They read transcripts independently and, along with a third analyst (the first author), developed codes in an iterative process. We combined a deductive and inductive approach to our analysis, guided by both the socioecological domains and our judgement as experienced practitioners. Both interviewers/analysts analyzed the first four transcripts (one from each of the four provider groups) and shared initial coding with the third reviewer. All co-authors met to discuss initial findings and develop preliminary codes which followed the interview protocol. Codes included factors in each socioecological domain that influenced linkage-making—for example “knowledge base” in the Individual domain. Each coder then coded another three transcripts, adding and refining codes. We came to an agreement about which passages best embodied each code and added them to the codebook. Through co-coding and consensus, we finalized a codebook which was then used to analyze remaining interviews.

Next, we applied the codes to the data, identifying text that embodied each code and identifying quotes. Supervised by the first author, the interviewers compared quotes for accuracy and discussed discrepancies until they achieved 100% agreement. We began to detect saturation or repetition of themes after analyzing 15 interviews. We learned that participants provided similar narratives, despite degrees of change in frequencies of linkages or changes in collaboration. Therefore, conducting cross-group analysis would have been futile. Codes captured concepts found across all interviews. In order to add rigor, we presented preliminary results to our Implementation Community Collaborative Board, a body of researchers and providers that oversees all procedures regarding this study [8,9]. The board reviewed the results and provided feedback and “member checking” [44].

## 3. Results

### 3.1. Sample Characteristics

We recruited five providers (*n* = 20) in each category: high and low frequency of linkages and high and low interprofessional collaboration. The sample included 14 females and six males. Ten providers identified as African American; seven White; two providers identified as Asian; six providers identified as Latinos; and one provider identified as “more than one race.” The mean age was 43 years (range = 27–66). Twelve providers had master’s degrees, four had bachelor’s degrees, three had high school diplomas, and one had an associate’s degree. Six providers were supervisors (e.g., of counselors, case managers, and outreach staff); two were counseling staff; four were case managers; four were educators/outreach workers; three were program administrators; and one was a health navigator.

### 3.2. Factors that Influence Linkage-Making

Table 1 shows quotes from the interviews representing narratives of how specific factors, in each of four socioecological domains, influenced care continuum linkage-making.

### 3.3. Individual Domain: Professional Knowledge Base

Providers highlighted distinct enabling factors—formal education and on-the-job training—which influenced their overall capacity to make care continuum linkages. Some participants referred to specific courses that helped them better understand and thus engage in interprofessional and inter-agency collaboration. Likewise, they highlighted that their professional knowledge base improved with interactive forms of on-the-job trainings, webinars, and workshops focusing on interactions with colleagues that could enhance linkage-making. Also evident in the transcript of interviews is the role of formal education as a way to better understand and to practice interprofessional collaboration (“buying into the whole thing about collaboration”).

### 3.4. Relationship Domain: Interprofessional Collaboration

Providers offered narratives highlighting the importance of social and professional connections in linking patients to the “right” agency and/or service provider. They described using such strategies as collaborating tools to build personal connections with other agencies and to distinguish between helpful and unhelpful colleagues and/or agencies. They cited specific examples, such as case management meetings, which serve as a method for bringing people together to socialize and share information. The narratives also hinted at the fact that not all providers might be willing to share their “right” contacts with colleagues who may not reciprocate.

### 3.5. Community Domain: Providers’ Work-Related Changes

Agency-level changes are usually put into place in response to policy changes, thus this factor can also be placed in the Policy domain. We chose the Community domain to highlight changes in *community*-based agencies. Providers discussed how increased data collection (e.g., patient satisfaction) in their agencies negatively affected the time they had to find and to link patients to appropriate services. Job changes, such as promotions, were also cited as increasing different responsibilities that might compete with linkage-making. They described how less time due to volume of day-to-day work meant making fewer linkages. Most important perhaps was discussion about providers being dismissed by their agencies and the difficulties a provider may face in developing new partnerships with new staff. This lack of continuity in staffing and interagency collaborators further reduced capacity for linkage-making.

### 3.6. Policy Domain: Best Practices in a Competitive Environment

Provider narratives reflected the importance of providing services grounded in HIV prevention best practices, while also reflecting the pressures providers feel to retain clients. Participants felt pressure from their agencies and from funding sources to link patients to evidence-based services and to retain as many patients as possible. Participants described a competitive environment and a fear of losing patients to other agencies. This competition was viewed as a barrier because providers fear linking patients to services in agencies that will not engage in *mutual* linkage-making. Nonetheless, providers also described the need to go outside their agencies in order to promote the work that they do, thus suggesting that each agency might have unique ways of providing services and also of developing partnerships. We placed best practices here because they are codified in the form of agency policy.

## 4. Discussion

As discussed in the introduction, the extant literature shows myriad patient-level factors that influence care continuum linkage-making. However, provider-, agency-, and policy-level factors are not as well-known. Therefore, this study responded to a call for research to determine sociomedical factors that may influence linkage-making. By using a socioecological framework and a diverse group of providers in our sample, this study revealed factors that influence linkage-making across four domains of reference.

### 4.1. Individual Domain: Professional Knowledge Base

Factors in the Individual domain included formal education that stresses collaboration as a toll for improving patient access to services, and also in-agency interactive trainings for attaining the knowledge base needed to link patients to care continuum services. Therefore, interactive training and workshops are recommended to improve provider knowledge base, including biomedical aspects of HIV, evidence-based practices, and linkage-making strategies [34,35]. Interactive knowledge-building strategies can also yield professional contacts and help providers develop more trust with one another. All this can potentially decrease providers’ fear of losing patients, and thus improve linkage-making.

### 4.2. Relationship Domain: Interprofessional Collaboration

Within the Relationships domain, participants highlighted interprofessional collaboration as having potential to improve linkage-making. Providers reported work-related factors (changing job roles) which may influence how often they engage in linkage-making [27,28]. From the text of interviews, we learned that providers believe in training strategies that bring together workers from different agencies in order to develop deeper professional connections with individual providers, but also between the agencies for which they work. This type of strategy has potential to create sustainability around collaboration in that solid partnerships across agencies can help maintain appropriate levels of linkage-making even in the face of high staff turnover.

### 4.3. Community Domain: Providers’ Work-Related Changes

Factors in the Relationships and Community domains expand the existing literature [33] by highlighting how linkage-making is influenced simultaneously by work-related changes (i.e., at the provider and agency level) and interprofessional connections. Work-related changes need not be an impediment to linkage-making. For example, promotions within an agency may mean that an increasing number of more junior staff is being trained to link patients to care. However, for providers to successfully transition to new positions while maintaining optimum levels of care continuum linkage-making, agency administrators will need to provide training and allot time dedicated for such activities. Similarly, training on “how to” and time dedicated to building interprofessional contacts and subsequent collaborations may help abate the fear of losing patients discussed above.

### 4.4. Policy Domain: Best Practices in a Competitive Environment

Participants identified environmental pressures; it is challenging for providers to link their patients to evidence-based services while retaining patients within their agencies. Factors in the Policy domain suggest that providers fear losing their patients, which may inhibit linkage-making across care systems. Provider-level fear can be exacerbated by organizational pressure to retain patients, particularly in service environments containing multiple organizations of varying sizes and capacities vying for limited funding. Nonetheless, a competitive environment, viewed by participants as a barrier, could potentially enable better care if policy makers were to create incentives for agencies to engage in reciprocal linkage-making and to fully align their financial mandates with best HIV prevention practices.

### 4.5. Implications for Multi-Level Interventions and Future Research

We reported the most salient socioecological factors explored by participants. Based on these findings, we suggest interventions targeting provider-, interprofessional-, agency- and policy-level factors with the potential to improve linkage-making. By specifying these different levels of influence, this study helps to close a persistent gap in the care continuum literature. These findings suggest interventions to decrease specific barriers and to build on known facilitators of linkage-making at the provider, interprofessional, agency and policy levels. Our findings suggest that all factors may influence one another within and across domains. Therefore, we recommend interventions aimed at modifying different factors in all four domains to achieve maximum effect toward improving care continuum linkage-making.

We hypothesize that by modifying, through training, providers’ professional knowledge and interprofessional collaboration, future survey research will detect more active linkages to evidence-based services. Likewise, longitudinal studies will detect the specific effects of HIV prevention policies over time. Myriad other less salient factors might influence linkage-making; given the limited number of providers and their concentration in one city, our framework must not be generalized. Future research needs to focus on the distinct and cumulative effect of each factor identified. The diversity of roles in our sample (managers, counselors, navigators) reflects the roles played by providers around the world; we are thus confident that similar findings may be obtained in similar sociopolitical environments.

Our study did not test associations between providers’ linkage-making and the moderating effects of environmental challenges on providers’ capacities to make linkages. However, the qualitative portion of the parent mixed methods study helps to explain the quantitative aspect of the longitudinal study. The strength of the mixed method lies in our ability to use quantitative data (changes in levels of linkage-making and interprofessional collaboration over time) to select four different groups of providers characterized by different degrees of linkage-making and interprofessional collaboration. This would not have been possible without the quantitative surveys we used at baseline and 12-month follow-up.

Many of the factors we identified in all domains of reference are themselves influenced by the funding available to the organizations where the providers work. By the time the parent study began in 2012, environmental and historical changes were coming about. After the 2008 financial crisis, NYC agencies, like many other urban agencies across the US, lost federal, state, and local funding, and many providers lost their jobs or had their hours cut. This trend persisted for several years, including during the period when the data reported here were collected. The consolidation of many HIV programs has resulted in more agencies losing funds and resources. These historic shifts are not the subject of inquiry in this study; however, we bring them up to contextualize the environments where this study took place.

## 5. Conclusions

This paper highlights the critical role of interprofessional collaboration in helping patients to access and stay in each of the steps of the care continuum. Our data revealed salient factors across four domains of influence: knowledge base, life- and work-related changes, interprofessional collaboration, and best practices. We showed that these factors influenced the providers’ capacity to link patients to services in each step of the HIV care continuum, such as HIV testing, HIV care, and support services. These findings offer a conceptual and empirical basis for developing and testing interventions to help providers improve patients’ access and retention in care. We recommend that providers be trained in areas of knowledge, opinions and attitudes toward best practices, and social norms related to interprofessional collaboration [45,46,47]. Though case management and peer navigator interventions have improved access to care [17,18,19,20,21], we recommend training diverse providers to learn from one another. Interprofessional collaboration might facilitate linkage-making and strengthen shared commitment by providers for best practices in patient care, ultimately achieving patient access/retention in the HIV care continuum [48,49].

## Figures and Tables

**Table 1:** Factors that influence providers’ linkage-making to care continuum services.

**Individual Domain: Professional Knowledge Base and Life Changes**	I think by doing different trainings or webinars … bringing people together to talk about challenges, then brainstorming different resources, I think is helpful. Because you tend to only see things through your agency lens and have certain referrals or who you would go to. [Participant ID: A06P06]
	I think that organizing interactive workshops where you’re sitting down with people from other agencies to try and figure out how to [link patients] is a good idea. [A01P14]
	I’m working on my master’s. I am starting to buy into the whole thing about collaboration. There were some classes I took that it was evident collaboration was important, where instead of it being one [provider], sometimes you need an outside [provider] along with you to observe while you’re doing it and vice versa. [A03P08]
**Relationship Domain: Interprofessional Collaboration**	I said to everyone every time we have a case management meeting, “The [providers] in this room, we have a gold mine of resources. Every one of you have your own contact in every facility. Share that with your coworkers.” Because that’s the way to get in. We build—we know [providers] by name. They like that we know them by names. [A01P04]
	I know these places exist, but without personal connections to those places, it feels like a shot in the dark [A01P14]
	You actually collaborate with your colleagues to know what agencies work, what don’t work. [A18P19]
	That’s been helpful to develop relationships so when somebody answers the phone I know who it is. “Is that really the earliest appointment?” “Don’t you have anything on Wednesday?” I feel our clients get better treatment. [A01P09]
**Community Domain: Providers’ Work-Related Changes**	I feel like, before, I had less responsibility. I just had less responsibility, so I had more time to go more in-depth with different things. Whereas now, it’s more job responsibility and more out-of-work responsibility, has affected whether or not I’m focused on making referrals to people. [A01P14]
	I would say when certain contracts end, [providers] get laid off or dismissed, you take on extra duties. When you take on extra duties that does affect how you collaborate because you have so much more to concentrate on instead of actually doing the actual collaboration. The client can suffer. [A03P08]
	In the last six months, one of the biggest changes is this managed care kit that’s coming out where everybody has to begin to really not only get to know the other agencies but begin collaboration for services that we don’t offer, so it’s been a lot of work. [A17P16]
**Policy Domain: Best Practices in a Competitive Environment**	I just think it’s important to get outside of your own agency and promote how you can work collaboratively. Because I think people are worried that you are going to swoop in and steal their clients. And it is a legitimate fear. [A02P14]
	It’s a very competitive world, and a lot of agencies feel that if they collaborate they will lose clients. If people would just get out of that mind frame and think that we’re all here together for one goal and we’re able to collaborate. [A12P12]
	That’s in the back of our minds, that we would not be sending somebody out to a non-evidence-based intervention… we definitely would be thinking about that. [A01P01]
	Yeah, that’s important to me also, because the evidence-based referral will show me that it worked. [A18P19]
	I think it’s about setting up a spirit of collaboration and delineating who does what and how we can help each other, and less of an environment of “you’re going to steal my clients”. [A02P14]
